# Advanced Heart Failure Therapies in Neuromuscular Diseases

**DOI:** 10.1007/s11936-024-01046-2

**Published:** 2024-06-25

**Authors:** Arianne Clare Agdamag, Phoo Pwint Nandar, W. H. Wilson Tang

**Affiliations:** 1 Section of Advanced Heart Failure and Transplantation Medicine, Department of Cardiovascular Medicine, Miller Family Heart, Vascular and Thoracic Institute, Robert and Suzanne Tomsich, Cleveland Clinic, Cleveland, OH 44195, USA; 2 Section of Advanced Heart Failure, Department of Medicine, MetroHealth Medical Center, Cleveland, OH 44109, USA

**Keywords:** Heart failure, Cardiomyopathy, Arrhythmia, Neuromuscular disorders, Heart transplant, Left ventricular assist device

## Abstract

**Purpose of Review:**

The main objective of this review article is to discuss the prevalence, utilization, and outcomes associated with advanced heart failure therapies among patients with neuromuscular disorders.

**Recent Findings:**

Neuromuscular disorders often have multisystem involvement with a high prevalence of cardiovascular pathology. With the improvement in management of respiratory related complications, heart failure is now the leading cause of mortality in this patient population. Advanced heart failure therapies with durable left ventricular assist devices and heart transplantation have proven to be feasible and safe treatment options in selected patients.

**Summary:**

Management of neuromuscular disease involves multidisciplinary team involvement given the systemic nature of the disease. Early recognition and close monitoring of these patients will allow for timely initiation of advanced heart failure therapies that can lead to successful outcomes.

## Introduction

**Fig. 1:**
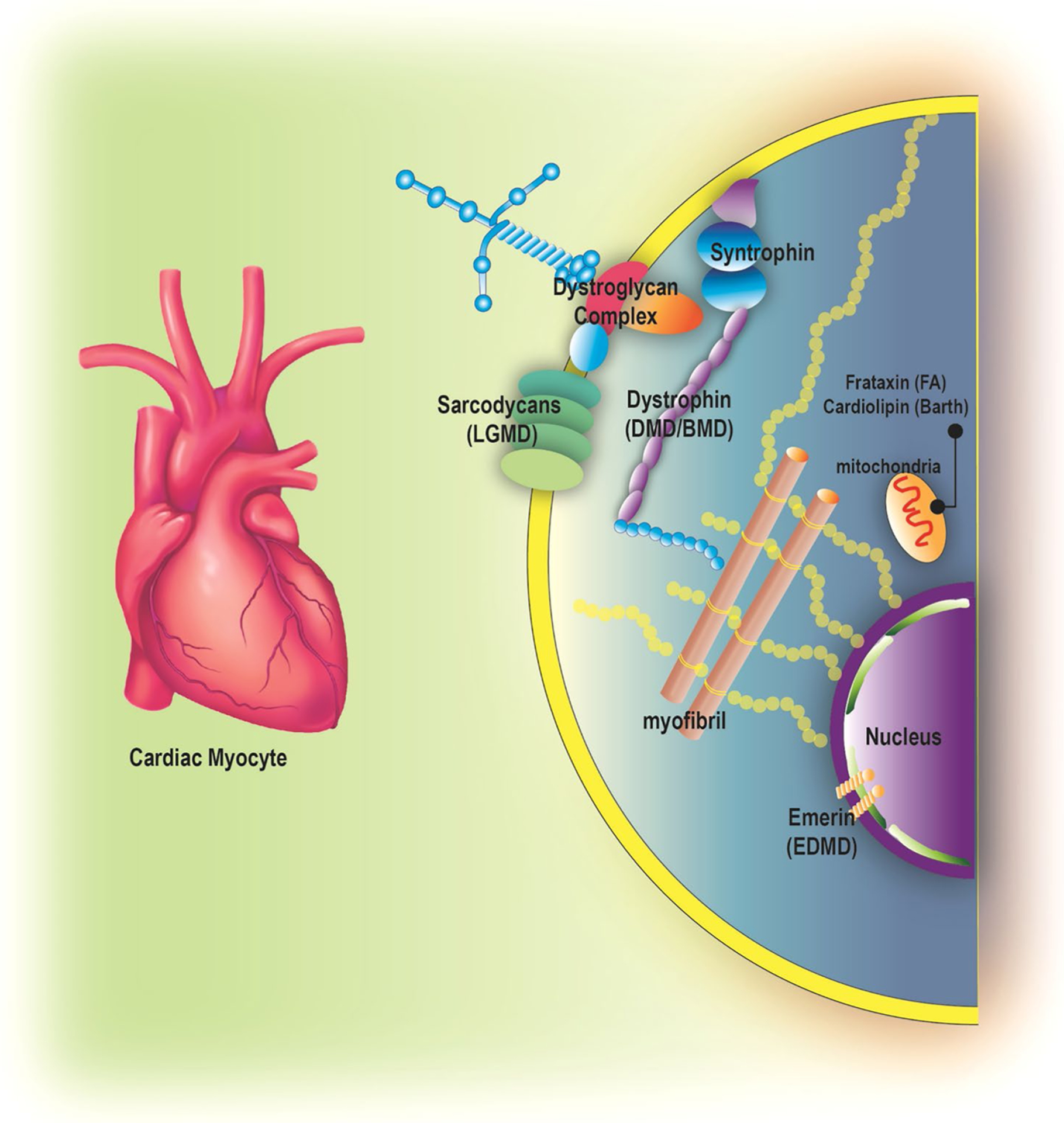
Proteins implicated in neuromuscular cardiomyopathy. LGMD, limb girdle muscular dystrophy; DMD, Duchenne muscular dystrophy; BMD Becker muscular dystrophy; FA, Friedrich ataxia; EDMD Emery-Dreifuss muscular dystrophy

**Fig. 2:**
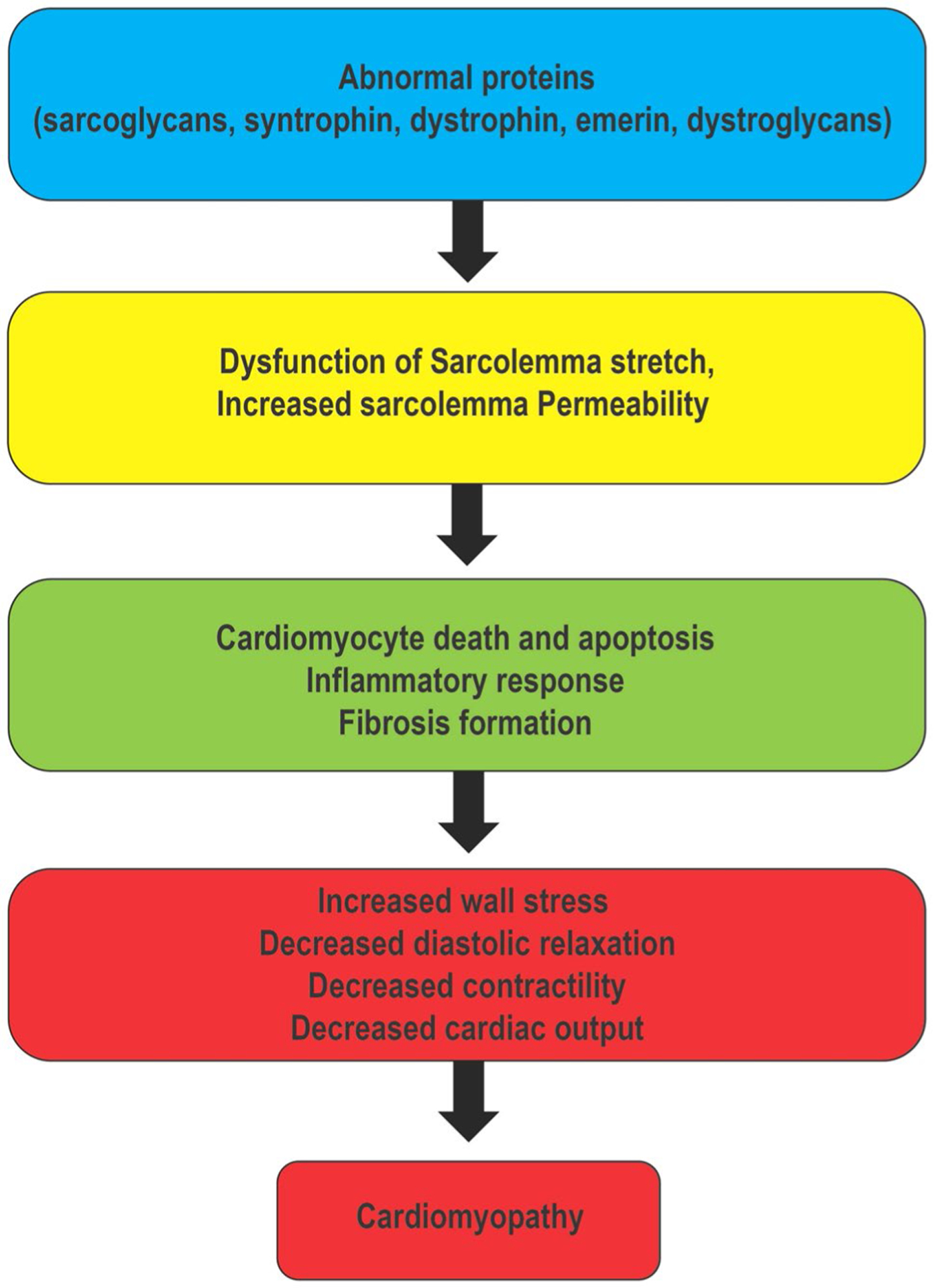
Pathophysiology of abnormality in neuromuscular protein leading to heart failure

Neuromuscular disorders (NMD) are a group of myogenic disorders which present in early childhood or adulthood with variable inheritance patterns, clinical symptoms, organ involvement, and prognosis [1]. NMD result from mutation and dysregulation of cytoskeletal or nuclear proteins which lead to progressive skeletal myopathies [2, 3]. Duchenne muscular dystrophy (DMD) and Becker muscular dystrophy (BMD) are the most common types of muscular dystrophy (MD). DMD and BMD have a high prevalence of cardiac involvement with mortality rates for heart failure at 40–50% [4–6]. Myotonic dystrophy (MYD), Limb-girdle muscular dystrophy (LGMD), Emery-Dreifuss muscular dystrophy (EDMD) and mitochondrial myopathies are rarer forms of NMD; however, these also have cardiac involvement. Multiple proteins have been implicated incardiac involvement in NMD (Fig. 1). These proteins affect cardiomyocytes and eventually lead to cardiomyopathy (Fig. 2). Cardiac manifestations of NMD vary and are summarized in Table 1. With the improvement in the management of respiratory related complications among NMD patients, heart failure is now the leading cause of mortality in this population [7, 8].

### Pathophysiology and Phenotypic Presentations of Neuromuscular Cardiomyopathies

#### Dystrophinopathies

Dystrophinopathies are X-linked muscle disorders resulting from mutations in the dystrophin gene (Xp21), causing abnormalities in the dystrophin protein crucial for maintaining muscle cell membrane integrity during contraction [9–13]. This leads to increased muscle fiber injury, and when repair is insufficient, necrotic myocytes are replaced by fibrofatty tissues, resulting in a spectrum of phenotypes with skeletal and cardiac muscle involvement [14–17].

Dystrophinopathic cardiomyopathy, resulting from dystrophin protein deficiency, progresses from a presymptomatic stage in childhood to overt cardiomyopathy with fibrosis and hypertrophy [12]. The incidence increases with age and by > 20 years old, nearly all patients have some sign of cardiac impairment [10]. As patients age, cardiac impairment increases, and end-stage heart failure becomes a leading cause of mortality, especially with improved respiratory symptom management in the current era [18, 19].

#### Dystrophinopathy—Duchenne Muscular Dystrophy

Duchenne muscular dystrophy (DMD) is the most common neuromuscular dystrophy affecting 1 in 3500 newborns. It has an early onset of clinical manifestation and has the most severe form of dystrophinopathy due to the complete absence of the dystrophin protein [8, 9]. The disease course has evolved, transitioning from a predominant respiratory focus to a cardiac etiology causing major complication [20]. The diagnosis of DMD is usually suspected based on the physical examination, family history, and laboratory tests (creatine kinase levels more than 100–200 times normal) and confirmed by genetic or immunohistochemical analysis [11, 21, 22]. EKG abnormalities are present in approximately half of patients (43% short PR, 37% RVH, 34% Q wave in V5, V6) with no relationship to the degree of cardiomyopathy. Moreover, the presence of Q waves was more often seen in the inferolateral leads than the anterior leads [23]. On echocardiogram, evidence of contractile and relaxation abnormalities as well as areas of akinesia or dyskinesia can be seen as the disease progresses [24–26]. Data suggests that boys with normal echocardiographic ejection fraction had fibrosis and decreased peak circumferential strain on MRI [26]. When compared with other etiologies of dilated cardiomyopathy, DMD cardiomyopathy has a higher mortality [10].

#### Dystrophinopathy—Becker Muscular Dystrophy

Becker muscular dystrophy (BMD) is a milder and less common dystrophinopathy affecting 1 in 18,450 male individuals [18]. Symptoms of BMD typically begin at around 11 years with a gradual onset of muscle weakness. Individuals with BMD may develop severe dilated cardiomyopathy potentially leading to irreversible heart failure [9]. Work-up includes genetic testing which has replaced invasive testing such as muscle biopsy. Genetic testing also provides information on benefits of specific therapies [27, 28]. BMD patients develop echocardiographic changes similar to DMD patients and cardiac MRI is the preferred imaging modality for early detection of cardiac dysfunction. There is no direct correlation between the severity of skeletal muscle involvement and the degree of dilated cardiomyopathy [29–33].

#### Myotonic Dystrophy

Myotonic dystrophy, an autosomal dominant disorder with CTG triplet repeat expansion in the myotonic dystrophy protein kinase gene, has two subtypes: DM1 and DM2. DM1, the more common form, is the primary adult-onset muscular dystrophy, while DM2, known as proximal myotonic myopathy, lacks DM1 molecular pathology [34–36]. The condition affects multiple organ systems, with the heart as the primary site of pathology, leading to cardiac manifestations, including arrhythmia, conduction disease, cardiomyopathy and mitral valve prolapse (13–40%), contributing to patient mortality [37, 38]. Echocardiographic findings can include diastolic dysfunction early in the disease progressing to systolic dysfunction. Regional wall motion abnormalities are also seen due to non-ischemic fibrosis [39]. MRI studies have shown fatty infiltration of the right ventricle in patients with ventricular tachyarrhythmias [40].

#### Limb Girdle Muscular Dystrophy

Limb Girdle Muscular Dystrophy (LGMD) is a heterogeneous group of disorders categorized as autosomal dominant (LGMD1) or autosomal recessive (LGMD2), initially presenting with proximal muscle weakness [41]. The autosomal recessive form exhibits progressive weakness, particularly in shoulder and pelvic muscles and may involve the heart, leading to various cardiac complications such as conduction disorders, arrhythmias, fatty infiltration, and cardiomyopathy [41, 42]. EKG, Echo and MRI findings are similar to DMD and BMD patients [43]. Incidence and age at presentation vary, with the lamin A/C subtype (LGMD1B) showing increased cardiac disease frequency, while calpain and dysferlin diseases have less cardiac involvement [44, 45]. The LMNA gene defect (lamin A/C subtype) can lead to adult-onset cardiomyopathy with minimal evidence of significant skeletal myopathy while some patients may progress to end-stage heart failure, requiring advanced heart failure therapies [46].

#### Emery-Dreifuss Muscular Dystrophy

Emery-Dreifuss Muscular Dystrophy is a nondystrophinopathy characterized by early onset joint contractures and slowly progressive muscle weakness [41]. Inheritance is X-linked, autosomal dominant or more rarely autosomal recessive. Cardiac involvement occurs in more than 90% of patients manifesting with conduction defects, arrhythmia and dilated cardiomyopathy [47, 48]. Given the high prevalence of cardiac disease in Emery-Dreifuss Muscular Dystrophy, the AHA consensus statement recommends annual ECG, echocardiography and ambulatory ECG for autosomal dominant and X-linked recessive EDMD and ECG and ambulatory ECG yearly for autosomal recessive EDMD [41].

#### Facioscapulohumeral Muscular Dystrophy

Facioscapulohumeral muscular dystrophy, the third most common type of muscular dystrophy, presents with symptoms in the second decade. Compared to BMD and DMD, it rarely has cardiac involvement [38].

#### Mitochondrial Myopathies

Mitochondrial myopathies, encephalomyopathies, and respiratory chain disorders result from mitochondrial or nuclear DNA abnormalities [49, 50]. The exact incidence of mitochondrial disease related cardiomyopathy is unknown. Cardiac involvement varies according to the type of mitochondrial disorder.

#### MELAS

Mitochondrial, Encephalopathy, Lactic Acidosis and Stroke like episodes (MELAS), the most common maternally inherited form, presents with ragged red fibers, diabetes, and renal issues [49]. Cardiac manifestations involve hypertrophic and dilated cardiomyopathy, characterized by abnormal left ventricular thickening progressing to severe dilation and poor contraction [51, 52].

#### MERFF

Myoclonus Epilepsy with Red Ragged Fibers (MERFF) manifests as myoclonus, seizures, ataxia, dementia, and skeletal muscle weakness. It is also typically associated with hypertrophic (symmetric or asymmetric) cardiomyopathy, dilated cardiomyopathy and less commonly arrhythmogenic histiocytoid cardiomyopathy [53].

#### Barth Syndrome

Barth syndrome is a rare X-linked mitochondrial disease which presents with hypotonia skeletal myopathy, growth delay, neutropenia, and increased urinary excretion of 3-methylglutaconic acid (3-MGCA) [54]. Cardiac manifestations include left ventricular noncompaction, endocardial fibroelastosis, hypertrophic or dilated cardiomyopathy, heart failure, and ventricular arrhythmia [55].

#### Kearnes-Sayre Syndrome

Kearnes-Sayre Syndrome is defined by the triad: onset before the age of 20, chronic progressive external ophthalmoplegia, and pigmentary retinopathy. Its cardiac manifestation determines prognosis [56]. These include conduction defects progressing to complete heart block, congestive heart failure, syncope, and sudden death. The mortality difference among those with no cardiac disease compared to those with cardiomyopathy is 26% vs 71% [56].

#### Friedreich’s Ataxia

Friedreich’s Ataxia, caused by frataxin gene alteration, results in central sensory pathway dysfunction, leading to progressive gait and limb ataxia, dysarthria, areflexia, decreased vibration sense, and muscle weakness [57]. Cardiac involvement includes LVH progression to systolic dysfunction and arrhythmia due to mitochondrial proliferation, loss of contractile proteins and myocardial fibrosis [57]. The degree of hypertrophy correlates with frataxin repeat length and GAA repeat size in smaller alleles [52, 53].

## Treatment Options

### Medical Management

Early-stage management of dystrophinopathic and mitochondrial cardiomyopathy involves guideline directed heart failure treatment (GDMT) with beta blocker, angiotensin converting enzyme inhibitor (ACE) or angiotensin receptor blocker (ARB), and mineralocorticoid receptor antagonist. Limited data exist regarding the potential benefits of angiotensin receptor neprilysin inhibitor or sodium glucose transporter 2 inhibitor in these patients as they have been excluded from clinical trials.

Among DMD patients, ACE inhibitors have been shown to be effective in slowing the onset of cardiomyopathy and has a noted mortality benefit [58]. Supplements such as coenzyme q-10 are added to minimize substrate deficiencies in the respiratory chain in patients with mitochondrial disease with the goal of improving cardiac symptoms; however, there are no randomized controlled trials to support this [59].

Apart from heart failure GDMT, the 2017 AHA Scientific Statement highlights the role of exercise, physical therapy, assisted ventilation, and palliative care in enhancing cardiac outcomes in this population [41]. The 2022 Consensus Statement from the Heart Rhythm Society (HRS) emphasizes that standard cardiomyopathy guidelines on cardiovascular implantable electronic device (CIED) implantation are also applicable in NMD patients. Condition specific technical challenges such as body habitus, respiratory muscle weakness, and sedation-related risks should also be considered in arrhythmia and CIED management. Oral anticoagulation for atrial fibrillation should be based on CHA2DS2-VASc score with the exception of patients with Emery-Dreifuss muscular dystrophy or limb-girdle muscular dystrophy type 1B due to the associated atrial standstill and elevated risk of thromboembolism [53]. Table 2 summarizes the AHA and HRS recommendations.

**Fig. 3:**
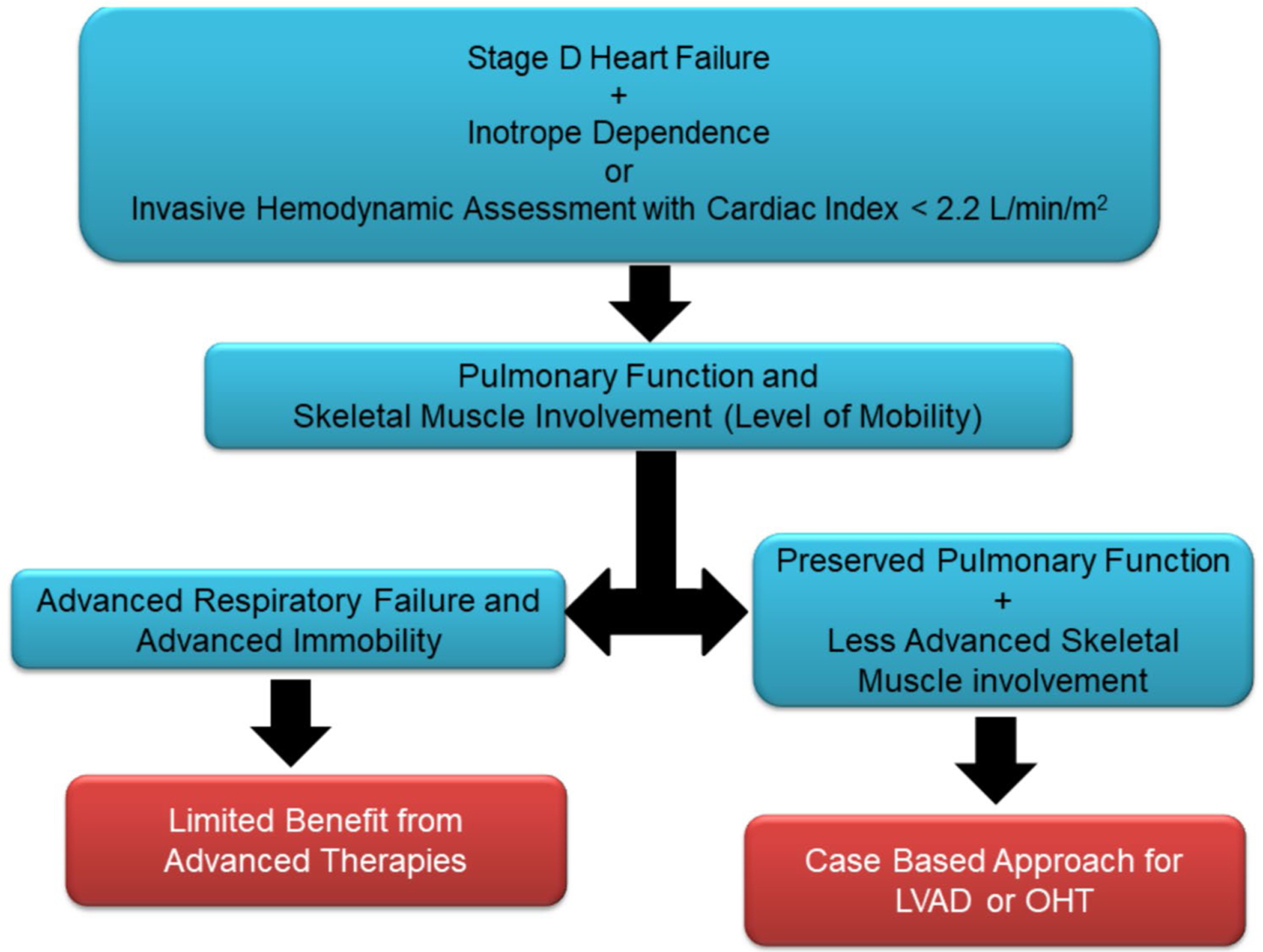
Proposed decision-making algorithm for advanced heart failure therapy in patients with neuromuscular dystrophy. LVAD – left ventricular assisted device, OHT – Orthotopic heart transplantation

Despite guideline directed therapy for heart failure, most patients can progress to end-stage heart failure requiring advanced therapies with either left ventricular assist devices or cardiac transplantation [35, 60, 61]. Selection of patients for advanced heart failure therapies should consider the severity of cardiac involvement, over-all prognosis, degree of skeletal muscle and respiratory system involvement [1]. Figure 3 includes a proposed algorithm for evaluating candidacy for advanced therapies among patients with NMD.

### Multidisciplinary Care for Neuromuscular Disease Patients

A multidisciplinary team led by a neurologist, with input from physical medicine and rehabilitation, cardiology, electrophysiology, pulmonary medicine, gastroenterology, endocrinology, orthopedic surgery, general surgery, and genetic counselor is needed to provide optimal care for NMD patients [53]. Given the multisystem involvement of the various disease entities, it is necessary to include all these subspecialties as part of the treatment team. Some centers have developed a Neuromuscular Cardiology team that partners with the Comprehensive Neuromuscular Center to streamline the evaluation of patients being considered for transplantation [1]. Due to the uniqueness of this patient population, there is a need to establish neuromuscular cardiomyopathy clinics that can provide regular cardiovascular visits, evaluation, and titration of medical therapies [62].

### Important Considerations for Advanced Heart Failure Therapy Evaluation

Neuromuscular disorders (NMDs), manifest at various ages and progress at differing rates, creating challenges in establishing uniform guidelines on assessing pulmonary function and skeletal muscle involvement as part of advanced heart failure evaluation.

Expert consensus recommends the use of pulmonary function testing (PFT), respiratory muscle strength testing and arterial blood gas analysis to aid in treatment decisions [63]. While impaired respiratory muscle function is not uncommon, this is critical for NMD with background genetic defect compromising baseline function and reserve signifying the concept of “reversible frailty”. Spirometry, lung volumes, and diffusing capacity of the lungs for carbon monoxide (DLCO) are essential for assessing pulmonary function, which typically correlate with the severity of respiratory muscle weakness, manifesting as restriction, and in cases of expiratory muscle weakness, reduced expiratory reserve volume (ERV) and increased residual volume (RV) with normal or elevated RV/TLC ratio. Vital capacity (VC) is unaffected by airflow obstruction and a decrease of over 30 percent in supine compared to upright posture suggests bilateral diaphragm weakness, potentially more reliably than maximal inspiratory pressure (PImax) [64, 65]. VC tends to fall late in progressive neuromuscular disease while the PImax falls earlier and correlates better with disease progression [66]. In cases where symptomatic NMD patients exhibit normal results in both PFT and overnight oximetry (ONO), the expert panel suggests that clinicians consider polysomnography to determine the clinical necessity of noninvasive ventilation (NIV) [63]. The respiratory muscle strength tests predict the prognosis and clinical consequences in progressive neuromuscular disease. A PImax below one-third of normal predicts hypercapnic respiratory failure (PaCO2 > 45 mmHg) and is linked to higher mortality rates [67, 68]. Sniff nasal inspiratory pressure (SNIP) measuring 35 percent of normal suggests a high likelihood of ventilatory failure in patients with neuromuscular disease [67, 69]. Maximal expiratory pressure (PEmax) less than 60 cm H_2_O predicts a weak cough with difficulty clearing secretions [70, 71]. Despite the clinical utility of these respiratory parameters, there are no specific cut-offs for each of these values in assessing candidacy for advanced therapies among patients with NMD.

Assessment for progressive skeletal muscle weakness and frailty involves a comprehensive history and physical examination. The Fried frailty assessment can be used as an objective assessment in patients with neuromuscular disorders [72]. Patient are evaluated for unintentional weight loss (> 5% of total body weight), grip strength measured with dynamometer (minimal values varies with sex and BMI), 15 feet walking speed (> 6–7 s), poor endurance and low energy expenditure (< 383 kcals per week for men and < 270 kcals per week for women) [72, 73]. Presence of these portend high risk features but are not absolute contraindications for advanced therapies.

An important consideration for patients about to undergo advanced heart failure therapies is the ability to participate in motor rehabilitation peri- and post-operatively. There is a potential for muscular damage at the time of surgery which can lead to lack of post-operative ambulatory recovery. Data shows that patients may incur mild muscle damage but majority of patients are able to ambulate independently post operatively [74, 75]. The exact etiology of ambulatory recovery after advanced HF therapies is unclear but may partially be due to recovery of disuse muscular atrophy from deconditioning while awaiting OHT or LVAD. Emphasis on pre-transplant rehabilitation monitored by physical therapists is critical in preventing further muscular deterioration and in promoting recovery after surgery. However, this has to be balanced with the susceptibility of NMD patients to excessive physical stress. Implementing patient-specific rehabilitation protocols and close monitoring of creatinine kinase levels may ameliorate ambulation capacity after surgery [75].

Cardiopulmonary exercise testing (CPET) is increasingly utilized to evaluate aerobic fitness in ambulatory patients with neuromuscular disease. It helps distinguish the dominant physiological system that limits exercise performance (cardiac, pulmonary, muscle metabolism or deconditioning) and aids in optimizing the therapeutic decision-making process [76]. There remains significant variation in standardizing and designing disease specific protocol with NMDs. Due to this, interpretation and application of results poses a challenge most especially in determining candidacy for advanced therapies. Currently, it is recommended to follow the American Thoracic Society and American College of Chest Physician recommendations on CPET protocol and interpretation [77].

### Orthotopic Heart Transplantation

Advanced heart failure therapy options for patients with MD are often limited due to lack of long-term studies reporting outcomes, provider reluctance, concern regarding comorbid conditions and possibility of development of cardiomyopathy in the transplanted heart [1, 60, 78]. The shortage of donor availability and the systemic nature of NMD also limit orthotopic heart transplantation (OHT) as an option for these patients [79]. However, more recent evidence have demonstrated that durable mechanical circulatory support devices and heart transplantation are feasible and valid therapies for NMD patients [1]. Prior registry data analyses demonstrated favorable survival results with OHT in NMD patients [1, 60]. Wu et al. analyzed the United Network for Organ Sharing UNOS database from 1990 to 2005 and identified 29 patients with NMD while Wells et al. expanded their analysis from 1987 to 2016 and identified 81 NMD patients who underwent OHT [1, 60]. In this larger cohort, the distribution of NMD are as follows: BMD 52%, EDMD 14%, LGMD 5%, DMD 3%, MYD 3%, Charcot-Marie-Tooth Dystrophy 1% and unspecified 22%. The median age of transplant is 22 years old (IQR 15–33). After propensity matching with patients listed for OHT due to idiopathic dilated cardiomyopathy, patients with MD had no significant difference in median time on waitlist, posttransplant length of hospital stay, stroke, dialysis incidence, and post-transplant survival. In fact, there was noted to be better survival in the MD cohort when compared to the cardiomyopathy-unmatched cohort (P = 0.004; HR (95% CI), 0.53 (0.34–0.82). These findings persisted with subgroup analysis comparing BMD vs non-BMD cohort [1].

There are currently no specific guidelines on listing criteria for patients with NMD. Candidates for OHT are often evaluated on a case-by-case basis and are listed for transplantation depending on the institution’s policies [9]. Centers that have transplanted NMD patients report favorable survival among these patients; however, they highlight the need for special care particularly in the peri- and post-operative period. These include appropriate close monitoring for neuromyological side effects from medications, dose adjustment of immunosuppressants in order to avoid secondary myopathy and rhabdomyolysis due to cyclosporine, monitoring for muscular toxicity with the combined use of lipid-lowering agents and cyclosporine, and continuation of steroids and NMD specific medication when applicable [9, 80].

With current data demonstrating similar long-term survival when compared to other patients, NMD patients who are good candidates should be considered for OHT. With heart failure being the leading cause of mortality, the long-term prognosis for these patients is closely linked to the feasibility of transplantation.

### Durable Mechanical Circulatory Support Devices

Durable left ventricular assist devices (LVAD) are utilized in patients with advanced heart failure who are not deemed to be candidates for heart transplantation [41]. There are no multi-center registry data that report the use of LVAD in patients with NMD. Table 3 summarizes published data documenting LVAD use in NMD.

Multiple centers have emphasized that the success of LVAD implantation relies on the involvement of a multidisciplinary team [81, 82]. Appropriate patient selection and optimization of functional status are of utmost importance to limit possible post-operative complications. NMD specific criteria that should be considered for patient selection include manual dexterity, handgrip strength, and functional capacity to operate the device and to perform battery or power source exchanges. Inclusion criteria include severe NYHA functional class IV and a legal guardian willing to comply for pediatric patients [81]. Exclusion criteria for LVAD implantation include: active sepsis, hepatic or renal dysfunction, coagulopathy or comorbidities limiting the use of anticoagulation, history of noncompliance, severe respiratory infection, severe right ventricular dysfunction, and life expectancy less than 1 year without HF [10, 82, 83]. The type of LVAD implanted has been varied in published literature. The Jarvik 2000 has been selected for certain pediatric patients because it is the only device with the possibility of positioning a power cable in the retroauricular position and has a lower infection risk compared to an abdominal cable [81].

Intra-operative considerations include the risk of rhabdomyolysis, malignant hyperthermia, and bleeding particularly with the interaction of the volatile anesthetics with muscle metabolism [84, 85].

Managing anesthesia for patients with muscular dystrophies presents significant challenges due to the increased risk of complications such as rhabdomyolysis leading to hyperkalemia and cardiac arrest, malignant arrhythmias, exacerbated muscle weakness, difficulties in airway management, and worsening respiratory failure. Total intravenous anesthesia is preferred for patients with muscular dystrophy. Volatile anesthetic agents are avoided due to their potential to induce myotonia and precipitate rhabdomyolysis crises [86]. In myotonic dystrophy, halogenated agents may increase muscular weakness [87, 88]. The most frequently used non-depolarizing muscle relaxant is rocuronium, which can be neutralized by a reversal agent [84]. Neostigmine can trigger acute myotony, rhabdomyolysis, malignant arrhythmias, and heart failure [88, 89]. Patients with muscular dystrophy have a high risk of apnea and death following extubation, particularly in the first 24 h after surgery [90]. If non-depolarizing muscle relaxants are necessary, it is crucial to achieve complete reversal to prevent any lingering muscle relaxation [90, 91].

The post-operative care after LVAD implantation should be taken with great consideration given the respiratory insufficiency and other systemic involvement in NMD patients [10, 83, 92]. Strong emphasis is given to aggressive pulmonary toilet and extubation to noninvasive positive pressure ventilation due to difficulty weaning from mechanical ventilation [81–83]. Other considerations include chest tube placement given the severe deformity and abnormally elevated diaphragm in the setting of severe kyphoscoliosis [10]. Early initiation of physical therapy to increase mobility, optimizing nutritional support, appropriate anticoagulation, and evaluation of psychosocial issues should be done shortly after stabilization [82, 83]. Preconditioning cycle with levosimendan has been demonstrated to improve clinical outcome in certain institutions [81]. Follow-up procedures include a monthly cardiac evaluation, complete cardiac ultrasound examination routine blood tests, EKG [81]. After hospital discharge, some institutions require discharge to temporary housing in close proximity to the hospital to foster independence in device care [93].

Long-term follow-up is needed with emphasis on maintenance of device functionality and preservation of ambulation and skeletal muscle strength. It has been demonstrated that new-onset obesity after LVAD implantation contributed to a decline in functional ability and increased incidence of falls which eventually lead to prolonged immobility and further loss of function [94].

Due to the limited data available, long-term outcomes are not well described in this population; however, the prior mentioned case series and case reports have reported that LVAD can be safely implemented and can improve survival and quality of life. Cantarutti et al. reported that LVAD implantation and concomitant use of neurohormonal blockade drugs lead to improvement in LVEF suggesting unloading of the left ventricle without a significant change in LV mass [95].

The Advanced Cardiac Therapies Improving Outcomes Network (ACTION) quality improvement collaborative is a working group of clinicians, family representatives, and patients that have put forth shared best practices around the use of LVAD particularly in the NMD patients. The ACTION network also aims to better describe LVAD outcomes in this population [83]. Further studies and bigger registry data are needed to evaluate the long term implications of LVAD implantation in this population as more patients are being implemented with LVAD for destination therapy [96].

### Emerging Therapies

**Fig. 4:**
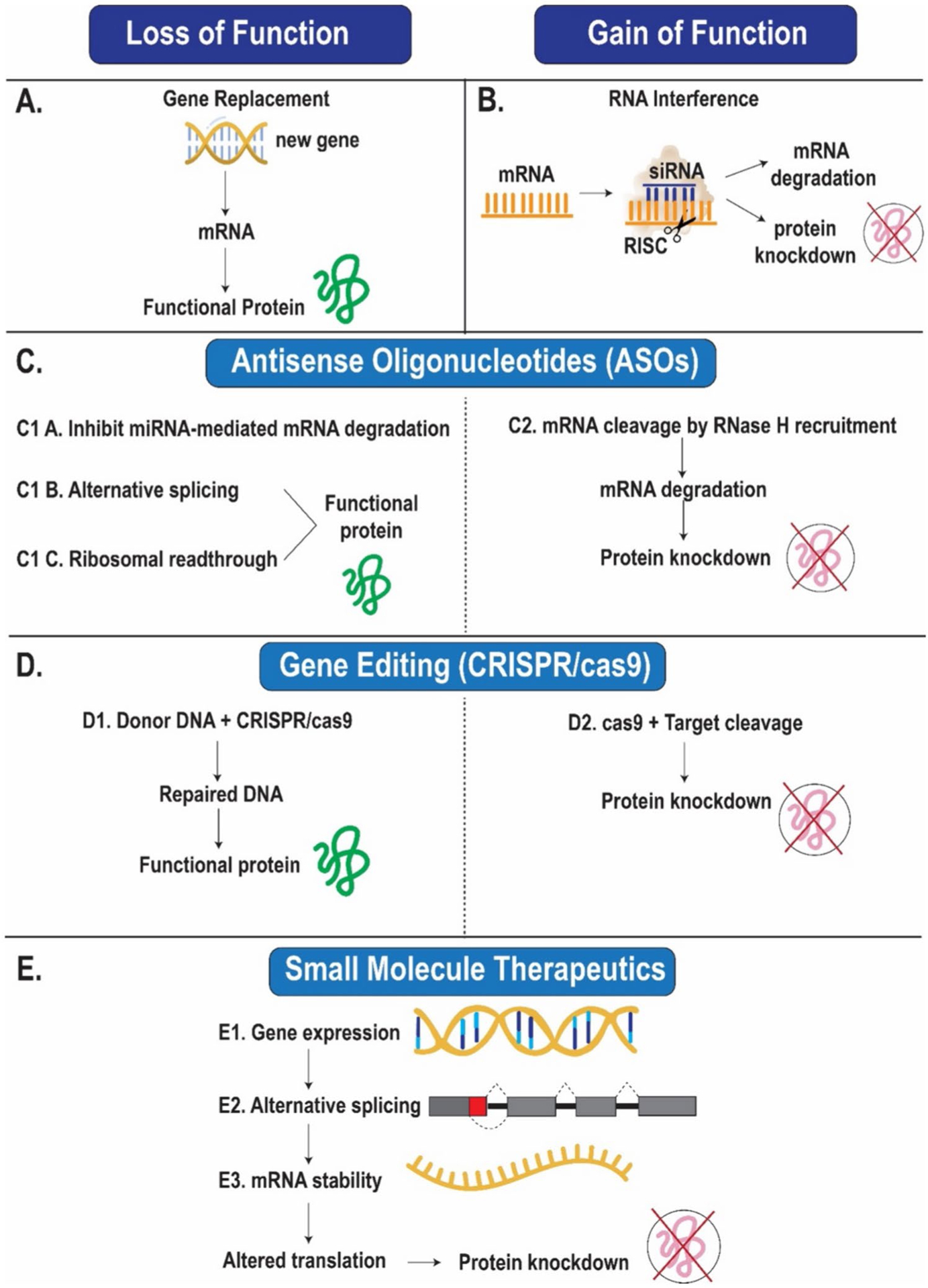
Summary of mechanisms of different gene therapies. **A-B** Gene replacement can lead to loss of function or gain of function. **C-D** ASO and CRISPR Cas9 can lead to functional proteins or protein knockdown. **E** Small molecule therapeutics can lead to protein knockdown. miRNA-microRNA; mRNA -messenger RNA; RISC – RNA induced silencing complex; siRNA – small interfering RNA

Gene therapy is an emerging therapeutic option for patients with NMD (Fig. 4). Gene therapy can be done with a) gene replacement which involves providing a functional copy of a missing or abnormal gene, b) gene editing which restores gene function by correcting a mutation or modification of gene expression, c) genetic therapies which modify the function of existing genes without permanently changing the gene [97]. Gene therapy is delivered via viral and nonviral methods. The main difference between the two vectors is the immunogenic potential of viral vectors. Adeno-associated viruses are the most used viral vectors in gene therapy trials due to their transduction efficiency and tropism for neurons and muscle [97, 98]. Toxicities with the use of AAV are immune related and can manifest as hepatotoxicity, acquired hemolytic uremic syndrome, neurotoxicity, and myocarditis. These manifest several weeks after gene therapy administration and can be resolved with corticosteroid treatment, eculizumab, plasmapheresis, or hemodialysis [99, 100].

Vitolarsen is an anti-sense oligonucleotide exon skipping therapy that works by shifting the reading frame to convert a DMD out-of-frame variant to an in-frame variant. Vitolarsen is a genetic therapy that modifies gene expression in vivo without changing the genome. Treatment with vitolarsen has demonstrated increased dystrophin production and improvement in timed function tests. It has been approved by the FDA in 2020. Apart from vitolarsen, there are 3 other genetic therapies approved for Becker and Duchenne Muscular dystrophy including eteplirsen, golodirsen, and casimersen. Gene therapy with FDA approval for SMA include nusinersen, onasemnogene, risdiplam [97].

There are multiple ongoing trials using gene therapy for spinal muscular atrophy, Becker and Duchenne muscular dystrophy, myotubular myopathy, limb-girdle muscular dystrophy, and Friedrich Ataxia. Most of the preliminary outcomes being investigated are related to the degree of protein expression, adverse events, and ambulatory improvement. There are some studies that included cardiac related secondary endpoints such as changes in ECG, echocardiography and CMRI findings. The data on these secondary endpoints have not been reported yet [98].

Gene therapy is a promising treatment option for NMD patients; however, the elevated cost, burden of administration, complications requiring prolonged duration of immunosuppression must be weighed against the potential benefits. Recent reported data on complications of gene therapy highlight the need for further investigation and refinement of this therapy. As more data become available and as the drug becomes more accessible and safe, hopefully, more patients will be able to derive its benefits.

## Conclusion

Neuromuscular disease patients have varying manifestations and severity of cardiac involvement. Published data have demonstrated that orthotopic heart transplantation is a safe and feasible option for appropriately selected candidates. Durable mechanical circulatory support devices have also been implanted as destination therapy for patients who are not deemed to be candidates for heart transplantation. There is a paucity in available data describing utilization of advanced heart failure therapies in this population signifying the need for more multicenter and non-registry data to describe the long-term outcomes. Gene therapies are a promising therapeutic option that can hopefully lead to systemic improvement alleviating the need for transplantation and durable mechanical circulatory support device implantation.

## Figures and Tables

**Table 1:** Cardiac Manifestation of NMD

Neuromuscular disease	Genetic Defect	Inheritance patterns	Demographics	Non-cardiac manifestation	Cardiac manifestation	Average age of onset of cardiac manifestation	Proportion of cardiac Manifestation
Duchenne muscular dystrophy	Dystrophin	X-linked recessive	Male, early age	Fatigue, muscle weakness, frequent falls, progressive difficulty in walking, learning difficulties, mental retardation	Abnormal EKG, Dilated Cardiomyopathy	6 years onwards	Abnormal EKG > 90% Abnormal Echo > 90% Cardiac Death 30–40%
Becker muscular dystrophy	Dystrophin	X-linked recessive	Male, adult	Gradual onset muscle weakness	Abnormal EKG, Myocardial Hypertrophy, Dilated Cardiomyopathy	20–40 years of age (can be disproportionate to muscular involvement)	Abnormal EKG >90% Abnormal Echo 65% Cardiac Death ~ 50%
Facioscapulo-humeral muscular dystrophy*	DUX4	Autosomal dominant	Any age Male = Female	Weakness of facial muscles, scapula stabilizers and dorsiflexors of the foot	Rare cardiac involvement Diastolic dysfunction Ventricular arrhythmia, and supraventricular arrhythmia	Adult	Focal myocardial fibrosis, fatty infiltration on MRI ~ 38% Arrhythmia ~ 23% Sudden cardiac death
Myotonic dystrophy (DM 1 -Steinert’s disease and DM2-PROMM)	DMPK ZNF9	Autosomal dominant	20–25 years old	Adult-onset myopathy, multiorgan involvement	Arrhythmia, conduction disorder (infra-His defect), cardiomyopathy, mitral valve prolapse	Young adult	Arrhythmia and conduction disorder ~ 90% Sudden cardiac death 30%
Limb Girdle muscular dystrophy (Sarcoglycano-pathies)	LGMD2C LGMD2D LGMD2E LGMD2F LGMD1B LGMD2I	Autosomal recessive and autosomal dominant	Male = Female	Progressive proximal muscle weakness	Conduction disorder, arrhythmia, fibrofatty infiltration, cardiomyopathy	Variable	20–25%
Emery-Dreifuss muscular dystrophy	EDMD1 EDMD2 EDMD3 LGMD1B	X-linked recessive Autosomal recessive, Autosomal dominant	Young male	Early onset joint contractures, Slowly progressive muscle weakness	Atrial arrhythmia (45%), Non sustained ventricular tachycardia (13%) AV block (37%), Dilated cardiomyopathy	10–39 years of age	> 95% by the age of 30 years
Myoclonus Epilepsy with Red Ragged (MERFF)	MT-TK gene	Maternally inherited mitochondrial myopathy	Early childhood or adolescence Male = Female	Myoclonus, seizure, ataxia, dementia, skeletal muscle weakness	Hypertrophic cardiomyopathy (asymmetrical septal hypertrophy), Dilated cardiomyopathy, Arrhythmogenic histiocytoid cardiomyopathy	30 –45 years of age	Limited data
Barth syndrome	Tafazzin	X-linked mitochondrial disease (female carriers are not affected)	Young males First year of life	Hypotonia, skeletal myopathy, growth delay, neutropenia, increased urinary 3-MGCA	LV non-compaction, fibroelastosis, Hypertrophic cardiomyopathy, Dilated cardiomyopathy, Ventricular arrhythmia	Heart failure in infancy or early childhood	Cardiomyopathy and ventricular arrhythmia ~ 90%
Kearnes-Sayre syndrome (very rare)	MTTL1	Large scale deletion of mitochondrial DNA	Young adult (onset ~ 20 year) 1.6 case per 100,000 in Finnish population	Muscle weakness, progressive external ophthalmoplegia, pigmentary retinopathy	Conduction defect (high degree AV block), Cardiomyopathy	Limited data	Limited data
Friedreich’s Ataxia	Frataxin	Autosomal recessive	Less than 25 years of age White (Western European descent) male = female	Progressive gait and limb ataxia, dysarthria, lower-limb areflexia, decreased vibration sense, muscle weakness	Interventricular septal hypertrophy, progressive systolic dysfunction, Arrhythmia (atrial fibrillation), heart block Myocardial fibrosis	Variable	60–70% LVH 45%

Key: *EKG* electrocardiogram, *Echo* echocardiogram, *MRI* magnetic resonance imaging, *DM 1*myotonic dystrophy type 1, *DM 2* myotonic dystrophy type 2, *PROMM* proximal myotonic myopathy, *LGMD* Limb Girdle Muscular Dystrophy, *EDMD* Emery Dreifuss Muscular Dystrophy, *3-MGCA* 3-methylglutaconic aciduria, *DNA* deoxyribonucleic acid, *LVH* left ventricular hypertrophy

**Table 2:** AHA and HRS Recommendations in patients with Neuromuscular Disease

	Heart Rhythm Society Consensus Statement [41]	American Heart Association Scientific Statement [53]
Baseline Cardiac Evaluation	Physical exam, ECG, ambulatory ECG, imaging (echocardiogram or cardiac MRI) even in the absence of symptoms	Physical exam, echocardiogram, ECG, ambulatory ECG, cardiac MRI in select patients
Monitoring	Periodic retesting recommended with serial imaging and ECG to monitor disease progression Repeat testing if symptoms develop or worsen	Every 6months - 1 year if with baseline abnormality Every 1–2 years if asymptomatic Repeat testing in the presence of new symptoms
Medical Therapy for Heart Failure	Follow heart failure guidelines to initiate GDMT when LVEF ≤ 40%	ACE inhibitors or ARB and Beta blockers in reduced LVEF MRA reasonable to consider in reduced LVEF Glucocorticoids if indicated for noncardiac conditions
Anticoagulation	For patients with atrial fibrillation, anticoagulation based in CHA_2_DS_2_-VAS_c_ and HAS-BLED In EDMD or LGMD Type 1B, treat with anticoagulation regardless of CHA2DS2-VASC	Anticoagulation indicated in the presence of atrial fibrillation or atrial flutter with other risk factors (HF, CVA, HTN) Aspirin and low dose anticoagulation in Barth syndrome and noncompaction phenotype
ICD/CRT-D	Defibrillator implant if LVEF ≤ 35% despite GDMT if concordant with patient’s goals In DM1 or DM2 requiring ICD, system with permanent pacing capability is recommended In EDMD or LGMD Type 1B, ICD is indicated in abnormal AV conduction even if asymptomatic In mitochondrial myopathies (ie. Kearns-Sayre syndrome), PPM implantation recommended in AV conduction abnormalities	Defibrillator implant follow standard guidelines. NYHA Class II/III and LVEF ≤ 35% In the setting of LMNA mutation, ICD instead of PPM is indicated regardless of LVEF If arrhythmia is a predominant feature (DMD, BMD, EDMD, DM1, FA, LGMD type 1B), ICD can be considered in select patients
Advanced Heart Failure Therapies	No mention	Durable mechanical support and cardiac transplant should be considered in carefully selected patients Home parenteral inotrope can be considered as part of palliative therapy
Palliative care	Early referral and consider patient’s goals of care during decision making	Early referral recommended
Other therapies	No mention	Exercise with submaximal effort strengthening regimens

* *ECG* electrocardiogram, *MRI* magnetic resonance imaging, *ACE* angiotensin converting enzyme, *ARB* angiotensin receptor blocker, *MRA* mineralocorticoid receptor antagonist, *LVEF* left ventricular ejection fraction, *EDMD* Emery Dreifuss muscular dystrophy, *LGMD* Limb girdle muscular dystrophy, *HF* heart failure, *CVA* cerebrovascular accident, *HTN* hypertension, *ICD* implantable cardiac defibrillator, *CRT-D* cardiac resynchronization therapy defibrillator, *GDMT* guideline directed medical therapy, *DM1* myotonic dystrophy type 1, *DM2* myotonic dystrophy type 2, *PPM* permanent pacemaker, *DMD* Duchenne muscular dystrophy, *BMD* Becker muscular dystrophy, *FA* Friedrich ataxia

**Table 3:** Case Reports and Case Series on the use of Durable Mechanical Circulatory Support Devices among patients with Neuromuscular Disease

Author	Type of NMD	Type of Device	Number of Patients	Average age at (years)	Years implanted	Outcomes
Perri et al. [10]	DMD (n = 6), Beta 2 sarcoglycan deficit (n = 1)	Jarvik 2000 VAD	7	16.5	2011–2016	3 deaths median follow-up time of 21.7 months
Stoller et al. [82]	DMD	Heartware	1	18	2013	Survived
Ryan et al. [93]	DMD	HM2, HW	2	26	2014	Survived
Wittlieb-Weber et al. [96]	DMD	HM2, HW	4	17	2016–2017	75% 1-year survival
Amodeo and Adorisio [101]	DMD	Jarvik 2000	2	15	2012	100% 6-month survival
Iodice et al. [81]	DMD	Jarvik 2000	4	12–17	2014	No long term outcome reported
Kim et al. [102]	DMD	HM3	1	20	2020	No long term outcome reported
Vishkin et al. [94]	BMD	HM2	2	15	2011–2012	100% survival in 6 months
Dedieu et al. [103]	Barth Syndrome	Berlin Heart EXCOR	1	3	2012	Bridged to transplant

* *DMD* Duchenne Muscular Dystrophy, *HM* HeartMate, *HW* HeartWare, *VAD* Ventricular Assist Device

## Data Availability

No datasets were generated or analysed during the current study.

## Opinion Statement

Management of neuromuscular disease involves a multi-disciplinary approach as these patients have complicated multi-organ involvement. Specialties including cardiology, cardiothoracic surgery, neurology, pulmonology, hematology, otolaryngology, orthopedic surgery, infectious diseases, general surgery, rehabilitation medicine, and palliative care are part of the care team. The prevalence of cardiac involvement varies depending on the type of neuromuscular disease. Imaging studies with transthoracic echocardiogram and cardiac magnetic resonance imaging allow for early detection of cardiomyopathy and can help guide treatment initiation and frequency of surveillance imaging. Early referral to an advanced heart failure specialist is often warranted since most patients progress to end stage heart failure and will require advanced therapies. Appropriate patient selection for durable left ventricular assist devices and orthotopic heart transplantation is critical due to evidence demonstrating survival benefits and improvement in quality of life in appropriately selected patients. Functional capacity, degree of skeletal muscle and respiratory involvement, life expectancy, and social support are some of the many factors considered during patient selection. Gene therapy has proven to be a promising treatment option in the field of neuromuscular disease. Currently, most of the endpoints reported have been related to improvement in functional capacity. However, upcoming trials have included cardiac outcomes as secondary endpoints.
